# Spine Fractures in Children and Adolescents—Frequency, Causes, Diagnostics, Therapy and Outcome—A STROBE-Compliant Retrospective Study at a Level 1 Trauma Centre in Central Europe

**DOI:** 10.3390/children8121127

**Published:** 2021-12-03

**Authors:** Stephan Payr, Andrea Schuller, Theresia Dangl, Britta Chocholka, Harald Binder, Thomas M. Tiefenboeck

**Affiliations:** Department of Orthopedics and Trauma Surgery, Division of Trauma Surgery, Medical University of Vienna, 1090 Vienna, Austria; stephan.payr@meduniwien.ac.at (S.P.); n1617891@students.meduniwien.ac.at (A.S.); theresia.dangl@meduniwien.ac.at (T.D.); britta.chocholka@meduniwien.ac.at (B.C.); harald.binder@meduniwien.ac.at (H.B.)

**Keywords:** spine fractures, paediatric trauma, epidemiology

## Abstract

The aim of this study was to present the frequencies and characteristics of paediatric spine fractures, focusing on injury mechanisms, diagnostics, management, and outcomes. This retrospective, epidemiological study evaluated all patients aged 0 to 18 years with spine fractures that were treated at a level 1 trauma centre between January 2002 and December 2019. The study population included 144 patients (mean age 14.5 ± 3.7 years; 40.3% female and 59.7% male), with a total of 269 fractures. Common injury mechanisms included fall from height injuries (45.8%), with an increasing prevalence of sport incidents (29.9%) and a decreasing prevalence of road incidents (20.8%). The most common localisation was the thoracic spine (43.1%), followed by the lumbar spine (38.2%), and the cervical spine (11.8%). Initially, 5.6% of patients had neurological deficits, which remained postoperatively in 4.2% of patients. Most (75.0%) of the patients were treated conservatively, although 25.0% were treated surgically. A small proportion, 3.5%, of patients presented postoperative complications. The present study emphasises the rarity of spinal fractures in children and adolescents and shows that cervical spine fractures are more frequent in older children, occurring with a higher rate in sport incidents. Over the last few years, a decrease in road incidents and an increase in sport incidents in paediatric spine fractures has been observed.

## 1. Introduction

Paediatric spine fractures are relatively rare, with an incidence ranging from 1% to 4% [1]. Currently, cervical spine fractures constitute about 1% of all spine fractures in children and adolescents, whereas thoracic and lumbar fractures make up 2–3% [2,3,4,5,6]. The localisation of spine fractures varies with age: thoracic and lumbar spine fractures occur more often in older children (>10 years), whereas cervical spine fractures occur more frequently in younger children [6,7,8]. The upper cervical spine (C0–C2) is especially at risk of fracturing in children younger than 8–10 years due to the different fulcrum and relatively large head compared to adults [9]. In addition, the paediatric spine is generally more unstable due to ligamentous laxity, weak paravertebral muscles, and the horizontal orientation of the facet joints [9]. At ages between 8 and 10 years, the anatomy and biomechanics of the paediatric spine are comparable with the adult spine [10].

Common causes for paediatric spine fractures are falls, sport and road incidents, as well as child abuse [2]; most epidemiological studies have identified road incidents are the most frequent cause of spine fractures [11,12]. Currently, epidemiological studies assessing the total spine (cervical, thoracic, and lumbar spine) are relatively rare; most studies either focus only on a special region of the spine, or present heterogenous injuries by also including ligamentous injuries. This makes current studies difficult to compare.

Due to the rarity of up-to-date epidemiological studies and their heterogeneity at present, the aim of this study was to describe the frequency and characteristics of paediatric spine fractures of the entire vertebral column, focusing on injury mechanisms, diagnostic procedures, management, and outcomes at a level 1 trauma centre.

## 2. Methods

This study was performed as a retrospective, epidemiological data analysis at a level I trauma centre and was approved by the Ethics Committee of the Medical University of Vienna (Code 1816/2020).

This study was performed following the STROBE guidelines in Appendix A.

**Figure 1:**
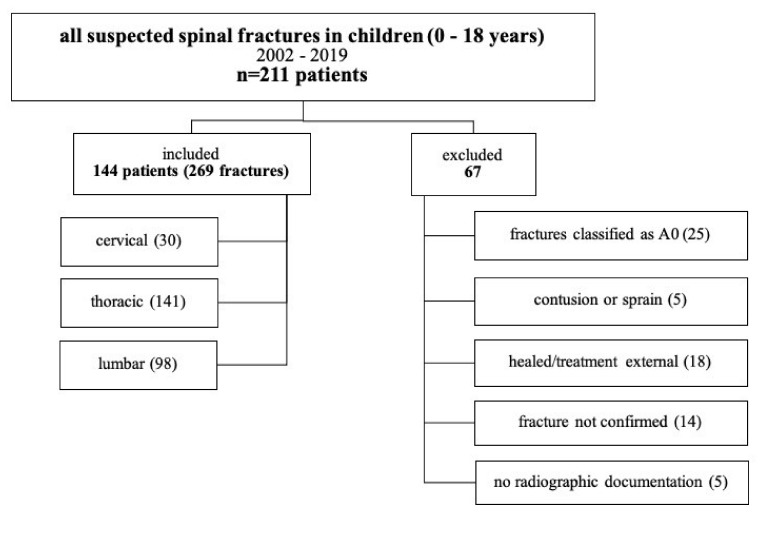
Flow chart of the overall study population (A0 = minor non-structural fractures; MRI, morphologically detectable “bone bruise” as well as spinous and transverse process fractures).

Initially, 211 children and adolescents aged from 0 to 18 years with spine fractures were treated at the Department of Trauma and Orthopaedic Surgery at the Medical University of Vienna, during an observation period from January 2002 to December 2019. Finally, 144 children were included after applying the inclusion and exclusion criteria (Figure 1).

All patients aged from 0 to 18 years with a fracture of the cervical, thoracic, and/or lumbar vertebra during the observation period were included. Exclusion criteria were age over 18 years, spinal injuries such as contusion or distortion, exclusive ligamentous injuries, sacral fractures, and fractures of the spinous or transverse processes, and the vertebral arch. Furthermore, healed or questionable fractures were excluded, as were patients who were initially treated at an external hospital. If radiological documentation was incomplete, patients were also excluded from the study.

The data were collected retrospectively from the patient’s charts, and included age, sex, injury mechanism, fracture localisation (cervical, thoracic, lumbar, or multiple regions), diagnostics using plain radiography (X-ray), computer tomography (CT scans), magnetic resonance imaging (MRI), management (operative or non-operative), as well as the exact surgical procedure or conservative treatment. Fractures were classified using the Gehweiler classification for C1 fractures, the Anderson and D’Alonzo classification for odontoid fractures, the Effendi classification for C2 fractures, and the AO Spine classification for lower cervical spine, thoracic, and lumbar spine fractures [13,14,15,16]. The clinical outcome, complications, and mobility (walking, crutches, bedridden, etc.) were extracted after treatment. Furthermore, the Frankel Score [17] was used to describe neurological deficits at the time of presentation and at the last follow-up.

### Data Analysis

Descriptive data are reported for the entire patient cohort, including the mean, range, and standard deviation (SD). In order to develop an epidemiological overview, the following parameters were evaluated: age, sex, fracture classification and localisation, injury mechanism, diagnostic imaging methods applied, management (operative or non-operative), surgical procedure, conservative treatment, neurological examination, complications, and mobility. Nominal and ordinal variables are presented as absolute and relative frequencies. Metric variables are reported as the mean, range, and standard deviation. The confidence interval for relative frequencies was 95%. Statistical analysis was performed using Microsoft Excel (Version 16.50., Microsoft Corp., Redmond, WA, USA) and SPSS software (Version 27.0.0., SPSS Inc., Chicago, IL, USA).

## 3. Results

In total, 144 patients aged 1 to 18 years (male: 86/144, 59.7%; female: 58/144, 40.3%; mean age, 14.5 ± 3.7 years) with spine fractures were included (Table 1). Of all patients, 73 (50.7%) sustained multiple fractures in more than one vertebral body, resulting in 269 fractured vertebrae in total. The mean follow-up time (FUP) was 12.9 ± 20.4 months, and the total mortality rate was 1.4%.

For the patient cohort, the following age groups were defined: 1.4% (2/144) toddlers (0 to 1 years); 3.5% (5/144) pre-schoolers (2 to 5 years); 9.7% (14/144) children in elementary school (6 to 11 years); 32.6% (47/144) high schoolers (12 to 15 years); and 52.8% (76/144) adolescents (16 to 18 years). The distribution of age is shown in Table 2.

### 3.1. Injury Mechanisms

**Figure 2:**
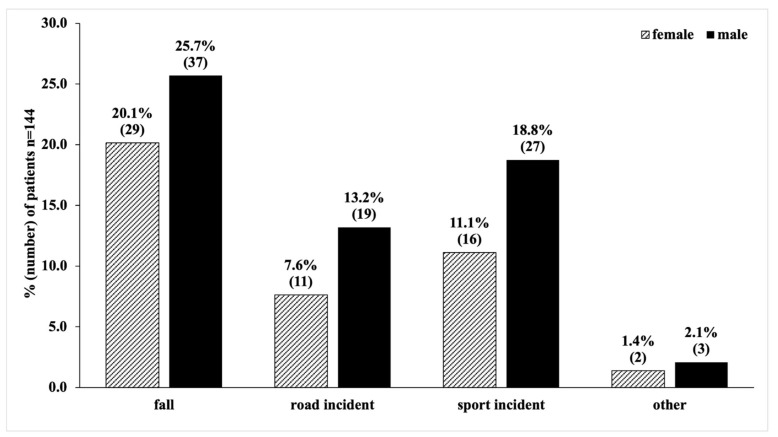
Distribution of injury mechanisms causing paediatric spine fractures.

Paediatric spine fractures were caused by fall from height injuries (66/144, 45.8%), sport incidents (43/144, 29.9%), road incidents (30/144, 20.8%), and other causes (5/144, 3.5%) (Figure 2).

Road incidents with motor vehicles included incidents as a driver or passenger and collisions between motor vehicles and pedestrians. Skiing (9/43, 21.0%) was the most common sport in which there were spinal injuries. In 10 patients (6.9%) (male: 5/144, 3.5%; female: 5/144, 3.5%; mean age 16.2 ± 1.2 years), attempted suicide, particularly by jumping from heights of more than three metres, was the cause of their injuries. The number of road incidents decreased, and the number of sport incidents increased during the observation period from 2002 to 2019, as shown in Table 3.

### 3.2. Fracture Characteristics and Management

**Figure 3:**
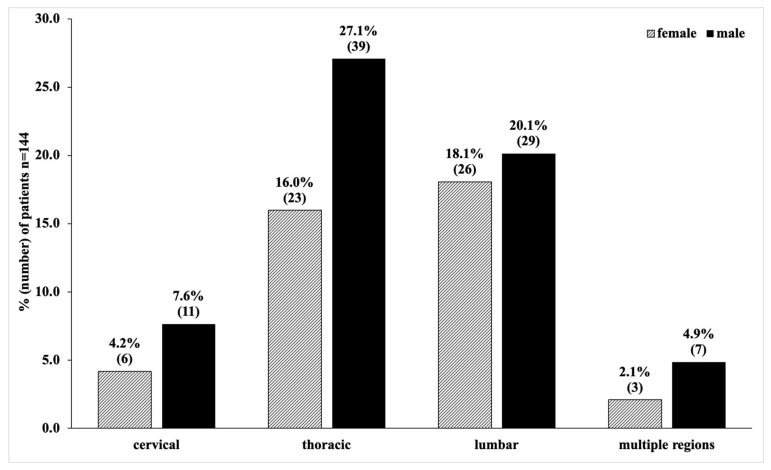
Distribution of fracture localization of paediatric spine fractures.

The thoracic spine (62/144, 43.1%) was the most frequently observed fracture localisation, followed by the lumbar spine (55/144, 38.2%), and the cervical spine (17/144, 11.8%) (Figure 3).

In 44 patients, fractures were located in the thoracolumbar (Th12 and/or L1); L1 was the most frequently observed fractured vertebra.

In the upper cervical spine, the following fractures were reported: 0.7% (2/269) odontoid fractures (Anderson and D’Alonzo type III); 1.1% (3/269) C1 fractures (2 Gehweiler type I, 1 type II); and 0.4% (1/269) C2 fracture (Effendi type I).

The most commonly observed fracture type in the lower cervical, the thoracic, and the lumbar spine was A1 (195/269, 72.5%). Severe fractures, such as type B (7/269, 2.6%) and type C (3/269, 1.1%), were rare, and every patient presenting with them was treated surgically. The majority of minor fractures, i.e., A1 and A2 fractures, were treated conservatively (74.3%, 200/269) (Table 4).

**Figure 4:**
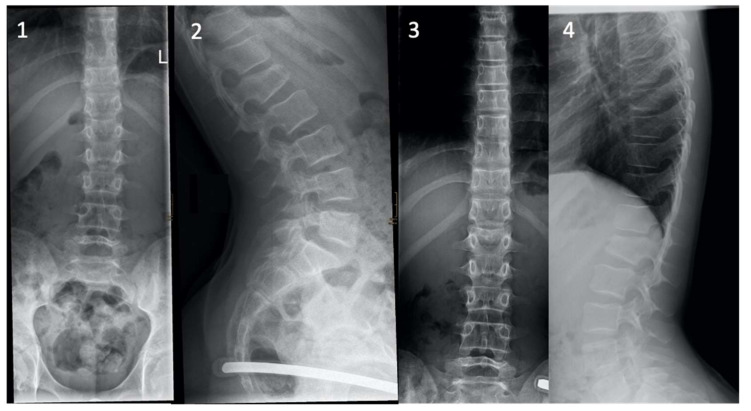
Images **1** and **2** show the radiographs of an A1.2 L1 fracture in a 12-year-old girl sustained after falling off a trampoline. The patient was treated conservatively by receiving adequate analgesia and sports abstinence. The radiographs at the last follow-up after three months of therapy show no further dynamics and a healed fracture (Images **3** and **4**).

Overall, 75.0% (108/144; mean age 14.2 ± 4.0 years) of paediatric spine fractures were treated conservatively. In the cervical region, hard collars (8/144, 5.6%), pain management and mobilisation (4/144, 2.8%), and one halo fixator (1/144, 0.7%) were applied. Conservative treatments of thoracic and lumbar fractures consisted of pain management, mobilisation (49/144, 34.0%), and bracing (46/144, 31.9%) (Figure 4).

**Figure 5:**
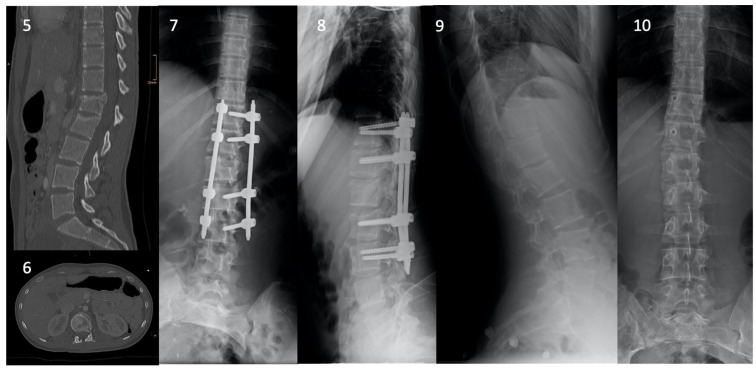
Images **5** and **6** illustrate a sagittal and axial CT scan of a 15-year-old girl with an A3.3 L1 fracture and an A3.1 L2 fracture after a suicide attempt by jumping from a bridge (height > 3 m). Images **7** and **8** show post-operative radiographs of the posterior stabilisation. Images **9** and **10** are the last radiographs obtained after implant removal (7 months post-operative), showing a fully consolidated fracture.

Surgical treatment was mostly indicated because of compression of the spinal canal (25/144, 17.4%) and was necessary in 25.0% (36/144) of patients. Anterior stabilisation was used in all patients with cervical spine fractures (6/144, 4.2%), with posterior stabilisation more frequently applied in the thoracic (7/144, 4.9%) and lumbar spine (10/144, 6.9%) (Figure 5).

### 3.3. Neurological Deficits and Outcome

In total, 5.6% (8/144; mean age 15.6 ± 4.7 years) of patients presented with neurological deficits after trauma and appeared most frequently in adolescents (7/8) and those who sustained sport incidents (3/8). Neurological involvement improved in two patients after treatment: one patient improved from Frankel A to Frankel C categorisation after posterior stabilisation, and one patient showed improvements after bracing from Frankel D to E. The other six patients (4.2%; mean age 15 ± 5.4 years) showed consistent neurological deficits after treatment.

Prior to trauma, no mobility restrictions were known in the entire patient cohort. After trauma and treatment, the mobility of the patients was reported at the FUP as follows: good walking ability (100/144, 69.4%), walking on crutches (2/144, 1.4%), using a walker (1/144, 0.7%), mobilised in a wheelchair (2/144, 1.4%), and bedridden (2/144, 1.4%). In 25.0% (36/144) of patients, the mobility documentation was missing.

Postoperative complications were classified according to the Clavien-Dindo Classification and occurred in 3.5% of patients (5/144), including wound infections, insufficient implants, breakage of screws, and screws invading the spinal canal. One complication (cage loosening, treated conservatively) was classified as group 1, and the other four complications requiring revisions under general anaesthesia were accordingly classified as group 3b. Patients who underwent a conservative treatment had no complications.

## 4. Discussion

The current study shows that paediatric spine fractures are relatively rare with a peak in middle aged children with a mortality rate of 1.4%. Similar data can be seen in many other retrospective studies [11,18,19,20]. In the current study, injury patterns changed from road incidents to sport incidents during the observation period. Accordingly, a higher frequency of cervical spine fractures was noted in adolescents. These findings are comparable to Poorman et al. and Shin et al., but are contrary to the findings of Compagnon et al. and Mahan et al., who reported a tendency of cervical spine injuries in younger children [18,21,22,23]. This discrepancy may be explained by differences in the study population and inclusion criteria. Poorman et al. only referred to cervical spine fractures in their study reporting a higher prevalence of cervical spine fractures in adolescents (ages 11–18 years) and young adults (ages 19–20 years) [21]. The inclusion criteria of Compagnon et al. and Mahan et al. contained not only vertebral fractures, but also ligamentous injuries of the paediatric cervical spine [18,23]. In addition, no differentiation was made between ligamentous injuries and solely bony fractures when it was stated that spinal injuries occurred, especially in young children (ages 0–8 years). It was not possible to determine the percentage of actual cervical spine fractures; hence, the findings cannot be compared directly to the current study. The reason for the higher frequency of younger children in these studies might be attributable to the inclusion of ligamentous injuries, because this is the main difference between the present study and that of Poorman et al. [21]. Therefore, cervical spine fractures were more frequently observed in high schoolers and adolescents. Furthermore, the higher rate of cervical spine fractures in high schoolers and adolescents in the present study may be related to the high frequency of sport incidents in this patient cohort. The results in the current study present sport incidents as the most frequent injury mechanism leading to cervical spine fractures (40% of all cervical patients; mean age 14.6 ± 3.8 years). High-schoolers (6) and adolescents (5), out of a total of 12 patients (91.7%), accounted for the majority of these patients, which is comparable to reports in the literature; the incidence of cervical spine injuries in sports increases with age [24]. Furthermore, the current data reveal that road incidents have decreased over the years: from 2002 to 2007, 16/44 (36.4%) patients suffered spinal fractures in a road incident, whereas from 2014 to 2019, the number decreased to 7/56 (12.5%) patients, which is in contrast to the extant literature [12,20,25,26]. However, the observation period of these studies is more comparable to the period from 2002 to 2007 than to the latest data (2014 to 2019). The decrease in road incidents may be related to the increased safety features of cars in the last few years, considering similar findings in a recent study from Compagnon et al. published in 2020 [18]. The posting of more speed limits may have influenced this trend as well. Although road incidents have decreased, we reported an increase in sport incidents, from 10 to 20 patients over the years, constituting 30% of all injury mechanisms. Similar findings have been presented in other retrospective studies [11,18,27,28]. Sport incidents might have become more frequent due to increases in at-risk sports such as horse riding [18] or skiing, the latter being the most frequently implicated sport in our study. A limitation of the present study is the retrospective design of our investigation and the relatively low number of patients, partly due to the fact that this was a single-centre study. However, the number of patients was also a result of the strict inclusion criteria necessary in order to generate a homogenous study population. We only included spine fractures classified from A1 to C (AO Spine Classification) and further excluded isolated injuries of the ligaments, because these are defined as minor injuries if still, stable conditions are present [29]. Furthermore, the injury pattern of SCIWORA (spinal cord injury without radiographic abnormality) was not included, because this study only focused on bony injuries. The lack of differentiation between ligamentous and osseous injuries in many studies resulted in a heterogeneity of injuries. The main advantage of the present study is the overview of paediatric spine fractures of a level one trauma centre and the strict inclusion criteria only including bony injuries to the spine. Additionally, the long observation period of almost 2 decades even enabled us to present changes in the frequencies of certain injury mechanisms.

## 5. Conclusions

The present study emphasises the rarity of spine fractures in children and adolescents and shows that cervical spine fractures are more frequent in older children, occurring with a higher rate in sport incidents. Over the last few years, a decrease in road incidents and an increase in sport incidents in paediatric spine fractures has been observed.

## Figures and Tables

**Table 1:** Demographic data.

*n* = 144		(%)	Mean Age	Standard Deviation	Age Range
				(SD)	
female (f)	58	40.3	14.2	4.0	1–18
male (m)	86	59.7	14.7	3.5	4–18
total	144	100.0	14.5	3.7	1–18

**Table 2:** Age groups.

Age Group (Years)	f	(%)	m	(%)	Total	(%)
toddler (0–1)	2	1.4	0	0	2	1.4
pre-schooler (2–5)	2	1.4	3	2.1	5	3.5
elementary (6–11)	5	3.5	9	6.3	14	9.7
high-schooler (12–15)	22	15.3	25	17.4	47	32.6
adolescent (16–18)	27	31.3	49	34	76	52.8

**Table 3:** Distribution of injury mechanisms from 2002 to 2019.

	2002–2007	(%)	2008–2013	(%)	2014–2019	(%)
Fall	16	11.1	24	16.7	26	18.1
Road Incident	16	11.1	7	4.9	7	4.9
Sports Incident	10	6.9	13	9.0	20	13.9

**Table 4:** AO Spine trauma classification system distributed between surgical (surg.) and conservative (cons.) treatment.

*n* = 269 Fractures									
Upper Cervical Spine	Total	Mean Age	(%)	Surg.	Mean Age	(%)	Cons.	Mean Age	(%)
Gehweiler Type I	2	2.5	0.7	0	-	0.0	2	2.5	0.7
Gehweiler Type II	1	4	0.4	0	-	0.0	1	4	0.4
Gehweiler Type III	0	-	0.0	0	-	0.0	0	-	0.0
Gehweiler Type IV	0	-	0.0	0	-	0.0	0	-	0.0
Gehweiler Type V	0	-	0.0	0	-	0.0	0	-	0.0
Anderson and D’Alonzo Type I	0	-	0.0	0	-	0.0	0	-	0.0
Anderson and D’Alonzo Type II	0	-	0.0	0	-	0.0	0	-	0.0
Anderson and D’Alonzo Type III	2	16.5	0.7	1	18	0.4	1	15	0.4
Effendi Type I	1	16	0.4	0	-	0.0	1	16	0.4
Effendi Type II	0	-	0.0	0	-	0.0	0	-	0.0
Effendi Type III	0	-	0.0	0	-	0.0	0	-	0.0
**Lower Cervical Spine**	**Total**	**Mean Age**	**(%)**	**Surg.**	**Mean Age**	**(%)**	**Cons.**	**Mean Age**	**(%)**
A1	15	16	5.6	4	17.3	1.5	11	15.5	4.1
A2	3	14.7	1.1	1	14.0	0.4	2	16.0	0.7
A3	3	17.3	1.1	2	17	0.7	1	18	0.4
A4	1	15.0	0.4	1	15.0	0.4	0	-	0.0
B1	0	-	0.0	0	-	0.0	0	-	0.0
B2	1	18.0	0.4	1	18.0	0.4	0	-	0.0
B3	0	-	0.0	0	-	0.0	0	-	0.0
C	1	17.0	0.4	1	17.0	0.4	0		0.0
**Thoracic Spine**	**Total**	**Mean Age**	**(%)**	**Surg.**	**Mean Age**	**(%)**	**Cons.**	**Mean Age**	**(%)**
A1	117	14.4	43.5	0	-	0.0	117	14.4	43.5
A2	3	15.7	1.1	1	15.0	0.4	2	16.0	0.7
A3	6	16.0	2.2	5	16.4	1.9	1	14.0	0.4
A4	8	15.4	3.0	4	15.3	1.5	4	15.5	1.5
B1	0	-	0.0	0	-	0.0	0	-	0.0
B2	5	8.4	1.9	5	8.4	1.9	0	-	0.0
B3	1	16.0	0.4	1	16.0	0.4	0	-	0.0
C	1	17.0	0.4	1	17.0	0.4	0	-	0.0
**Lumbar Spine**	**Total**	**Mean Age**	**(%)**	**Surg.**	**Mean Age**	**(%)**	**Cons.**	**Mean Age**	**(%)**
A1	63	14.5	23.4	1	15.0	0.4	62	14.5	23.0
A2	9	13.6	3.3	3	16.0	1.1	6	12.3	2.2
A3	10	15.9	3.7	7	16.1	2.6	3	15.3	1.1
A4	15	15.8	5.6	12	16.5	4.5	3	13.3	1.1
B1	0	-	0.0	0	-	0.0	0	-	0.0
B2	0	-	0.0	0	-	0.0	0	-	0.0
B3	0	-	0.0	0	-	0.0	0	-	0.0
C	1	16.0	0.4	1	16.0	0.4	0	-	0.0

## Data Availability

The datasets generated and/or analysed during the current study are not publicly available due to data privacy, but are available from the corresponding author upon reasonable request.

## Appendix A

**STROBE Statement**—checklist of items that should be included in reports of observational studies.

	**Item No**	**Recommendation**
Title and abstract (applied)	1	(*a*) Indicate the study’s design with a commonly used term in the title or the abstract
(applied)		(*b*) Provide in the abstract an informative and balanced summary of what was completed and what was found
**Introduction**		
Background/rationale (applied)	2	Explain the scientific background and rationale for the investigation being reported
Objectives (applied)	3	State specific objectives, including any prespecified hypotheses
**Methods**		
Study design (applied)	4	Present key elements of study design early in the paper
Setting (applied)	5	Describe the setting, locations, and relevant dates, including periods of recruitment, exposure, follow-up, and data collection
Participants (not applicable)	6	(*a*) *Cohort study*—Give the eligibility criteria, and the sources and methods of selection of participants. Describe methods of follow-up *Case-control study*—Give the eligibility criteria, and the sources and methods of case ascertainment and control selection. Give the rationale for the choice of cases and controls *Cross-sectional study*—Give the eligibility criteria, and the sources and methods of selection of participants
(not applicable)		(*b*) *Cohort study*—For matched studies, give matching criteria and number of exposed and unexposed *Case-control study*—For matched studies, give matching criteria and the number of controls per case
Variables (applied)	7	Clearly define all outcomes, exposures, predictors, potential confounders, and effect modifiers. Give diagnostic criteria, if applicable
Data sources/measurement (applied)	8 *	For each variable of interest, give sources of data and details of methods of assessment (measurement). Describe comparability of assessment methods if there is more than one group
Bias	9	Describe any efforts to address potential sources of bias
Study size (applied)	10	Explain how the study size was arrived at
Quantitative variables (applied)	11	Explain how quantitative variables were handled in the analyses. If applicable, describe which groupings were chosen and why
Statistical methods (applied)	12	(*a*) Describe all statistical methods, including those used to control for confounding
(not applicable)		(*b*) Describe any methods used to examine subgroups and interactions
(not applicable)		(*c*) Explain how missing data were addressed
(not applicable)		(*d*) *Cohort study*—If applicable, explain how loss to follow-up was addressed *Case-control study*—If applicable, explain how matching of cases and controls was addressed *Cross-sectional study*—If applicable, describe analytical methods taking account of sampling strategy
(not applicable)		(*e*) Describe any sensitivity analyses
**Results**		
Participants (applied)	13 *	(*a*) Report numbers of individuals at each stage of study—eg numbers potentially eligible, examined for eligibility, confirmed eligible, included in the study, completing follow-up, and analysed
(applied)		(*b*) Give reasons for non-participation at each stage
(applied)		(*c*) Consider use of a flow diagram
Descriptive data (applied)	14 *	(*a*) Give characteristics of study participants (eg demographic, clinical, social) and information on exposures and potential confounders
(applied)		(*b*) Indicate number of participants with missing data for each variable of interest
(applied)		(*c*) *Cohort study*—Summarise follow-up time (eg, average and total amount)
Outcome data (not applicable)	15 *	*Cohort study*—Report numbers of outcome events or summary measures over time
(not applicable)		*Case-control study—*Report numbers in each exposure category, or summary measures of exposure
(applied)		*Cross-sectional study—*Report numbers of outcome events or summary measures
Main results (not applicable)	16	(*a*) Give unadjusted estimates and, if applicable, confounder-adjusted estimates and their precision (eg, 95% confidence interval). Make clear which confounders were adjusted for and why they were included
(not applicable)		(*b*) Report category boundaries when continuous variables were categorized
(not applicable)		(*c*) If relevant, consider translating estimates of relative risk into absolute risk for a meaningful time period
Other analyses (not applicable)	17	Report other analyses performed—eg analyses of subgroups and interactions, and sensitivity analyses
**Discussion**		
Key results (applied)	18	Summarise key results with reference to study objectives
Limitations (applied)	19	Discuss limitations of the study, taking into account sources of potential bias or imprecision. Discuss both direction and magnitude of any potential bias
Interpretation (applied)	20	Give a cautious overall interpretation of results considering objectives, limitations, multiplicity of analyses, results from similar studies, and other relevant evidence
Generalisability (applied)	21	Discuss the generalisability (external validity) of the study results
**Other Information**		
Funding (not applicable)	22	Give the source of funding and the role of the funders for the present study and, if applicable, for the original study on which the present article is based
Note: An Explanation and Elaboration article discusses each checklist item and gives the methodological background and published examples of transparent reporting. The STROBE checklist is best used in conjunction with this article (freely available on the Web sites of PLoS Medicine at http://www.plosmedicine.org/, Annals of Internal Medicine at http://www.annals.org/, and Epidemiology at http://www.epidem.com/). Information on the STROBE Initiative is available at www.strobe-statement.org. * Give information separately for cases and controls in case-control studies and, if applicable, for exposed and unexposed groups in cohort and cross-sectional studies.		
